# *BAFF*, *APRIL* and *BAFFR* on the pathogenesis of Immunoglobulin-A vasculitis

**DOI:** 10.1038/s41598-021-91055-z

**Published:** 2021-06-01

**Authors:** Diana Prieto-Peña, Fernanda Genre, Sara Remuzgo-Martínez, Verónica Pulito-Cueto, Belén Atienza-Mateo, Javier Llorca, Belén Sevilla-Pérez, Norberto Ortego-Centeno, Leticia Lera-Gómez, María Teresa Leonardo, Ana Peñalba, Javier Narváez, Luis Martín-Penagos, Emilio Rodrigo, José A. Miranda-Filloy, Luis Caminal-Montero, Paz Collado, Javier Sánchez Pérez, Diego de Argila, Esteban Rubio, Manuel León Luque, Juan María Blanco-Madrigal, Eva Galíndez-Agirregoikoa, Oreste Gualillo, Javier Martín, Santos Castañeda, Ricardo Blanco, Miguel A. González-Gay, Raquel López-Mejías

**Affiliations:** 1 Research Group on Genetic Epidemiology and Atherosclerosis in Systemic Diseases and in Metabolic Bone Diseases of the Musculoskeletal System, IDIVAL, Division of Rheumatology, Hospital Universitario Marqués de Valdecilla, Avenida Cardenal Herrera Oria s/n, 39011 Santander, Spain; 2 `López Albo´ Post-Residency Programme, Hospital Universitario Marqués de Valdecilla, Santander, Spain; 3 Epidemiology and Computational Biology Department, School of Medicine, Universidad de Cantabria, and CIBER Epidemiología y Salud Pública (CIBERESP), Santander, Spain; 4 Division of Paediatrics, Hospital Universitario San Cecilio, Granada, Spain; 5 Medicine Department, Universidad de Granada, Granada, Spain; 6 Division of Paediatrics, Hospital Universitario Marqués de Valdecilla, Santander, Spain; 7 Division of Rheumatology, Hospital Universitario de Bellvitge, Barcelona, Spain; 8 Division of Nephrology, Hospital Universitario Marqués de Valdecilla, IDIVAL-REDINREN, Santander, Spain; 9 Division of Rheumatology, Hospital Universitario Lucus Augusti, Lugo, Spain; 10 Internal Medicine Dept, Hospital Universitario Central de Asturias, Instituto de Investigación Sanitaria del Principado de Asturias (ISPA), Oviedo, Spain; 11 Division of Rheumatology, Hospital Universitario Severo Ochoa, Madrid, Spain; 12 Division of Dermatology, Hospital Universitario de La Princesa, Madrid, Spain; 13 Division of Rheumatology, Hospital Universitario Virgen del Rocío, Sevilla, Spain; 14 Division of Rheumatology, Hospital Universitario de Basurto, Bilbao, Spain; 15 SERGAS (Servizo Galego de Saude) and IDIS (Instituto de Investigación Sanitaria de Santiago), NEIRID Lab (Neuroendocrine Interactions in Rheumatology and Inflammatory Diseases), Research Laboratory 9, Hospital Clínico Universitario de Santiago, Santiago de Compostela, Spain; 16 Instituto de Parasitología y Biomedicina ‘López-Neyra’, CSIC, PTS Granada, Granada, Spain; 17 Rheumatology Department, Hospital Universitario de La Princesa, IIS-Princesa, Madrid, Spain; 18 School of Medicine, Universidad de Cantabria, Santander, Spain; 19 Cardiovascular Pathophysiology and Genomics Research Unit, School of Physiology, Faculty of Health Sciences, University of the Witwatersrand, Johannesburg, South Africa

**Keywords:** Rheumatic diseases, Vasculitis syndromes

## Abstract

BAFF, APRIL and BAFF-R are key proteins involved in the development of B-lymphocytes and autoimmunity. Additionally, *BAFF, APRIL* and *BAFFR* polymorphisms were associated with immune-mediated conditions, being *BAFF* GCTGT>A a shared insertion-deletion genetic variant for several autoimmune diseases. Accordingly, we assessed whether *BAFF, APRIL* and *BAFFR* represent novel genetic risk factors for Immunoglobulin-A vasculitis (IgAV), a predominantly B-lymphocyte inflammatory condition. *BAFF* rs374039502, which colocalizes with *BAFF* GCTGT>A, and two tag variants within *APRIL* (rs11552708 and rs6608) and *BAFFR* (rs7290134 and rs77874543) were genotyped in 386 Caucasian IgAV patients and 806 matched healthy controls. No genotypes or alleles differences were observed between IgAV patients and controls when *BAFF, APRIL* and *BAFFR* variants were analysed independently. Likewise, no statistically significant differences were found in the genotype and allele frequencies of *BAFF, APRIL* or *BAFFR* when IgAV patients were stratified according to the age at disease onset or to the presence/absence of gastrointestinal (GI) or renal manifestations. Similar results were disclosed when *APRIL* and *BAFFR* haplotypes were compared between IgAV patients and controls and between IgAV patients stratified according to the clinical characteristics mentioned above. Our results suggest that *BAFF, APRIL* and *BAFFR* do not contribute to the genetic network underlying IgAV.

## Introduction

B cell-activating factor (BAFF, also known as B-lymphocyte stimulator or BlyS) and a proliferation-inducing ligand (APRIL) are cytokines expressed by antigen-presenting cells that play a crucial role in the development of B-lymphocytes^1–4^. BAFF receptor (BAFF-R) is the major mediator of BAFF-dependent costimulatory responses in circulating peripheral B-lymphocytes^5^, essential for its survival and maturation. Several pieces of evidence revealed that BAFF, APRIL and BAFF-R are molecules also involved in autoimmunity^6–8^. In this regard, an influence of *BAFF, APRIL* and *BAFFR* polymorphisms was observed on several immune-mediated conditions^9–11^, being *BAFF* GCTGT>A a shared insertion-deletion variant for multiple sclerosis, systemic lupus erythematosus (SLE), and rheumatoid arthritis^9,12^.

Immunoglobulin-A vasculitis (IgAV), formerly called Henoch-Schönlein purpura (HSP), is an inflammatory small-sized blood vessel disease, more common in children and rarer but more serious in adults^13–15^. The classic clinical triad of IgAV consists of palpable purpura, arthralgias/arthritis and gastrointestinal (GI) tract involvement. Renal manifestations are also common in affected patients and constitutes the most serious complication of the disease^16–18^. IgA1-predominant immune deposits in the vessel walls are the defining pathophysiologic feature of IgAV^13^, supporting the hypothesis that this vasculitis is predominantly a B-lymphocyte mediated disease. Furthermore, IgAV has a multifactorial aetiology in which genes play a relevant role in both the predisposition and severity of the disease^19–21^.

Taken all these considerations into account, this study aimed to determine, for the first time, whether *BAFF, APRIL* and *BAFFR* represents novel genetic risk factors for the pathogenesis of IgAV. For this purpose, *BAFF* rs374039502 polymorphism, which colocalizes with the *BAFF* GCTGT>A insertion-deletion variant mentioned above, and two tag polymorphisms within *APRIL* (rs11552708 and rs6608) and *BAFFR* (rs7290134 and rs77874543), which cover most of the variability of both genes, were genotyped in the largest series of Caucasian patients diagnosed with IgAV ever assessed for genetic studies.

## Patients and methods

### Study population

A series of 386 unrelated Spanish patients of European ancestry who fulfilled both Michel et al*.*^22^ and the American College of Rheumatology^23^ classification criteria for IgAV-HSP were included in the present study. Centres involved in the recruitment of these patients included Hospital Universitario Marqués de Valdecilla (Santander), Hospital Universitario San Cecilio (Granada), Hospital Universitario de Bellvitge (Barcelona), Hospital Universitario Lucus Augusti (Lugo), Hospital Universitario Central de Asturias (Oviedo), Hospital Universitario Severo Ochoa and Hospital Universitario de La Princesa (Madrid), Hospital Universitario Virgen del Rocío (Sevilla) and Hospital Universitario de Basurto (Bilbao). Information on the main clinical features of these patients is shown in Table 1. For GI manifestations, bowel angina was considered present if there was diffuse abdominal pain that worsened after meals, or bowel ischemia usually with bloody diarrhoea. GI bleeding was defined as the presence of melena, haematochezia, or a positive test for occult blood in the stool. Renal manifestations were defined to be present if at least one of the following findings was observed: haematuria, proteinuria, or nephrotic syndrome at any time over the clinical course of the disease and/or renal sequelae (persistent renal involvement) at last follow-up. With regard to treatment, glucocorticoids were used in patients with severe GI and/or renal manifestations. Mycophenolate or azathioprine were added to glucocorticoids in refractory patients. Cyclophosphamide and plasma exchange were required in two patients due to life-threatening manifestations.

**Table 1:** Main clinical features of the 386 patients with IgAV included in the study.

	% (n)
Children (age ≤ 20 years)/adults (age > 20 years) (n)	309/77
Percentage of females	47.9
Age at disease onset (years, median [IQR])	7 [5–19]
Duration of follow-up (years, median [IQR])	1 [1–3]
Palpable purpura and/or maculopapular rash	100 (386)
Arthralgia and/or arthritis	54.9 (212)
**GI manifestations (if “a” and/or “b”)**	53.6 (207)
a) Bowel angina	50.8 (196)
b) GI bleeding	17.1 (66)
**Renal manifestations (if any of the following characteristics)**	37.0 (143)
a) Haematuria^a^	35.5 (137)
b) Proteinuria^a^	33.7 (130)
c) Nephrotic syndrome^a^	5.7 (22)
d) Renal sequelae (persistent renal involvement)^b^	6.7 (26)

IgAV: IgA vasculitis; IQR: interquartile range; GI: gastrointestinal.

^a^At any time over the clinical course of the disease.

^b^At last follow-up.

In addition, a set of 806 sex and ethnically matched healthy controls without history of cutaneous vasculitis or any other autoimmune disease, constituted by blood donors from Hospital Universitario Marqués de Valdecilla (Santander) and National DNA Bank Repository (Salamanca), was also included in this study.

For experiments involving humans and the use of human blood samples, all the methods were carried out in accordance with the approved guidelines and regulations, according to the Declaration of Helsinki. All experimental protocols were approved by the Ethics Committees of Cantabria (for Hospital Universitario Marqués de Valdecilla, Santander), Ethics Committee of clinical research of Granada (for Hospital Universitario San Cecilio, Granada), Ethics Committee of clinical research of Barcelona (for Hospital Universitario de Bellvitge, Barcelona), Ethics Committee of clinical research of Galicia (for Hospital Universitario Lucus Augusti, Lugo), Ethics Committee of clinical research of Asturias (for Hospital Universitario Central de Asturias, Oviedo), Ethics Committee of clinical research of Madrid (for Hospital Universitario Severo Ochoa and Hospital Universitario de la Princesa, Madrid), Ethics Committee of clinical research of Sevilla (for Hospital Universitario Virgen del Rocío, Sevilla) and Ethics Committee of clinical research of Euskadi (for Hospital Universitario de Basurto, Bilbao). Informed written consent was obtained from all subjects.

### Single nucleotide polymorphisms selection and genotyping methods

Genomic deoxyribonucleic acid (DNA) from all the individuals was extracted from peripheral blood using REALPURE `SSS´ kit (RBME04, REAL, Durviz S.L., Spain).

Patients with IgAV and healthy controls were genotyped for the *BAFF* rs374039502 single nucleotide polymorphism using a custom TaqMan assay (ID: AH0JGPG) with the following primers: forward 5′-GACAGCATCCCGGTTTTCATTTTAT-3′ and reverse 5′-TGTAAACTGTTAAATGAAGTAAACAGTTAAAACTGA-3′. In addition, all individuals recruited in this study were genotyped for two tag genetic variants within *APRIL* (rs11552708 and rs6608) and two tag polymorphisms within *BAFFR* (rs7290134 and rs77874543), using predesigned TaqMan assays (C_25630192_20 for rs11552708, C_247220_20 for rs6608, C_2189968_1_ for rs7290134 and C_102764384_20 for rs77874543). Tagging of *APRIL* and *BAFFR* was performed using data from the 1000 Genomes Project (http://www.internationalgenome.org/) and the Haploview v4.2 software (http://broad.mit.edu/mpg/haploview), and considering the r^2^ threshold set at 0.8 and minimum minor allele frequency at 0.05. The linkage disequilibrium pattern of the *APRIL* and *BAFFR* polymorphisms analysed in this study is shown as Supplementary Figure 1 and Supplementary Figure 2 online, respectively.

Genotyping was performed in a QuantStudio™ 7 Flex Real-Time polymerase chain reaction system, according to the conditions recommended by the manufacturer (Applied Biosystems, Foster City, CA, USA).

Negative controls and duplicate samples were included to check the accuracy of the genotyping.

### Statistical analyses

All genotype data were checked for deviation from Hardy–Weinberg equilibrium (HWE).

Differences in genotype and allele frequencies of *BAFF, APRIL* and *BAFFR* as well as differences in haplotype frequencies of *APRIL* and *BAFFR* were evaluated between patients with IgAV and healthy controls and between patients with IgAV stratified according to specific clinical characteristics of the disease (age at disease onset or presence/absence of GI or renal manifestations).

First, comparisons were performed considering each *BAFF, APRIL* and *BAFFR* polymorphism independently. Both genotype and allele frequencies were calculated and compared between the groups mentioned above by chi-square or Fisher tests when necessary (expected values below 5). Strength of association was estimated using odds ratios (OR) and 95% confidence intervals (CI).

Subsequently, allelic combinations (haplotypes) of both *APRIL* and *BAFFR* polymorphisms were carried out. Haplotype frequencies were calculated by the Haploview v4.2 software (http://broad.mit.edu/mpg/haploview) and then compared between the groups mentioned above by chi-square or Fisher tests. Strength of association was estimated by OR and 95% CI.

P-values lower than 0.05 were considered as statistically significant.

All analyses were performed with the STATA statistical software 12/SE (Stata Corp., College Station, TX, USA).

## Results

The genotyping success rate for each polymorphism evaluated in this study was 99.4% for *BAFF* rs374039502 and *APRIL* rs11552708, 99.6% for *APRIL* rs6608, 99.7% for *BAFFR* rs7290134 and 99.3% for *BAFFR* rs77874543.

No evidence of departure from HWE was observed at the 5% significance level.

Genotype and allele frequencies of *BAFF, APRIL* and *BAFFR* variants were similar to those reported for populations of European origin in the 1000 Genomes Project (http://www.internationalgenome.org/).

### Differences in genotype and allele frequencies of *BAFF*, *APRIL* and *BAFFR* between patients with IgAV and healthy controls

Firstly, we compared genotype and allele frequencies of each *BAFF, APRIL* and *BAFFR* variant assessed independently between patients with IgAV and healthy controls.

As shown in Table 2, no statistically significant differences in *BAFF, APRIL* and *BAFFR* frequencies were disclosed when patients with IgAV were compared to healthy controls.

**Table 2:** Genotype and allele frequencies of *BAFF, APRIL* and *BAFFR* in patients with IgAV and healthy controls.

SNP	Locus	Change	Samples set	Genotypes, % (n)			Alleles, % (n)	
		1/2		1/1	1/2	2/2	1	2
rs374039502	*BAFF*	T/A	IgAV patients	91.9 (353)	8.1 (31)	0	95.9 (737)	4.1 (31)
			Healthy controls	91.5 (733)	8.1 (65)	0.4 (3)	95.6 (1531)	4.4 (71)
rs11552708	*APRIL*	G/A	IgAV patients	78.1 (299)	20.6 (79)	1.3 (5)	88.4 (677)	11.6 (89)
			Healthy controls	77.9 (625)	20.4 (164)	1.6 (13)	88.1 (1414)	11.9 (190)
rs6608	*APRIL*	C/T	IgAV patients	71.9 (277)	26.0 (100)	2.1 (8)	84.9 (654)	15.1 (116)
			Healthy controls	70.0 (561)	27.6 (221)	2.5 (20)	83.7 (1343)	16.3 (261)
rs7290134	*BAFFR*	A/G	IgAV patients	58.0 (224)	36.3 (140)	5.7 (22)	76.2 (588)	23.8 (184)
			Healthy controls	57.2 (459)	36.4 (292)	6.5 (52)	75.3 (1210)	24.6 (396)
rs77874543	*BAFFR*	G/C	IgAV patients	82.7 (316)	16.0 (61)	1.3 (5)	90.7 (693)	9.3 (71)
			Healthy controls	83.0 (666)	16.6 (133)	0.4 (3)	91.3 (1465)	8.7 (139)

IgAV: IgA vasculitis; SNP: single nucleotide polymorphism.

No statistically significant differences in *BAFF, APRIL* and *BAFFR* genotype and allele frequencies were disclosed when patients with IgAV were compared to healthy controls (p ≥ 0.05 in all the cases).

### Differences in genotype and allele frequencies of *BAFF*, *APRIL* and *BAFFR* between patients with IgAV stratified according to specific clinical characteristics of the disease

Subsequently, we compared genotype and allele frequencies of each *BAFF, APRIL* and *BAFFR* variant assessed independently between patients with IgAV stratified according to specific clinical characteristics of the disease.

Since IgAV is often a benign and self-limited pathology in children and a more severe condition in adults, we analysed potential differences in genotype and allele frequencies of *BAFF, APRIL* and *BAFFR* between patients with IgAV stratified according to the age at disease onset. However, no statistically significant differences in *BAFF, APRIL* and *BAFFR* frequencies were detected when children (age ≤ 20 years) were compared to adults (age > 20 years) (Table 3).

**Table 3:** Genotype and allele frequencies of *BAFF, APRIL* and *BAFFR* in patients with IgAV stratified according to specific clinical characteristics of the disease.

Polymorphism	Children (Age ≤ 20 years)		GI manifestations		Renal manifestations	
	Yes (n = 309)	No (n = 77)	Yes (n = 207)	No (n = 179)	Yes (n = 143)	No (n = 243)
***BAFF* rs374039502**						
TT	92.2 (284)	90.8 (69)	92.2 (190)	91.6 (163)	90.1 (128)	93.0 (225)
TA	7.8 (24)	9.2 (7)	7.8 (16)	8.4 (15)	9.9 (14)	7.0 (17)
AA	0	0	0	0	0	0
T	96.1 (592)	95.4 (145)	96.1 (396)	95.8 (341)	95.1 (270)	96.5 (467)
A	3.9 (24)	4.6 (7)	3.9 (16)	4.2 (15)	4.9 (14)	3.5 (17)
***APRIL* rs11552708**						
GG	78.1 (239)	77.9 (60)	76.7 (158)	79.7 (141)	81.1 (116)	76.3 (183)
GA	20.3 (62)	22.1 (17)	22.3 (46)	18.6 (33)	18.9 (27)	21.7 (52)
AA	1.6 (5)	0	1.0 (2)	1.7 (3)	0	2.1 (5)
G	88.2 (540)	89.0 (137)	87.9 (362)	89.0 (315)	90.6 (259)	87.1 (418)
A	11.8 (72)	11.0 (17)	12.1 (50)	11.0 (39)	9.4 (27)	12.9 (62)
***APRIL* rs6608**						
CC	70.8 (218)	76.6 (59)	69.6 (144)	74.7 (133)	75.5 (108)	69.8 (169)
CT	26.6 (82)	23.4 (18)	28.0 (58)	23.6 (42)	23.1 (33)	27.7 (67)
TT	2.6 (8)	0	2.4 (5)	1.7 (3)	1.4 (2)	2.5 (6)
C	84.1 (518)	88.3 (136)	83.6 (346)	86.5 (308)	87.1 (249)	83.7 (405)
T	15.9 (98)	11.7 (18)	16.4 (68)	13.5 (48)	12.9 (37)	16.3 (79)
***BAFFR* rs7290134**						
AA	58.9 (182)	54.5 (42)	55.0 (113)	62.0 (111)	60.1 (86)	56.8 (138)
AG	35.9 (111)	37.7 (29)	40.7 (85)	30.7 (55)	32.2 (46)	38.7 (94)
GG	5.2 (16)	7.8 (6)	4.3 (9)	7.3 (13)	7.7 (11)	4.5 (11)
A	76.9 (475)	73.4 (113)	75.1 (311)	77.4 (277)	76.2 (218)	76.1 (370)
G	23.1 (143)	26.6 (41)	24.9 (103)	22.6 (81)	23.8 (68)	23.9 (116)
***BAFFR* rs77874543**						
GG	83.0 (254)	81.6 (62)	81.6 (168)	84.1 (148)	83.1 (118)	82.5 (198)
GC	16.0 (49)	15.8 (12)	17.5 (36)	14.2 (25)	16.9 (24)	15.4 (37)
CC	1.0 (3)	2.6 (2)	1.0 (2)	1.7 (3)	0	2.1 (5)
G	91.0 (557)	89.5 (136)	90.3 (372)	91.2 (321)	91.5 (260)	90.2 (433)
C	9.0 (55)	10.5 (16)	9.7 (40)	8.8 (31)	8.5 (24)	9.8 (47)

IgAV: IgA vasculitis; GI: gastrointestinal.

No statistically significant differences in *BAFF, APRIL* and *BAFFR* genotype and allele frequencies were disclosed between patients with IgAV stratified according to the age at disease onset or the presence/absence of GI or renal manifestations (p ≥ 0.05 in all the cases).

We also examined whether differences in the genotype and allele frequencies of *BAFF, APRIL* and *BAFFR* could exist between patients with IgAV stratified according to the presence/absence of GI or renal manifestations. Accordingly, no statistically significant differences in *BAFF, APRIL* and *BAFFR* frequencies were found between patients with IgAV with or without GI manifestations (Table 3). This was also the case when patients with IgAV who developed renal manifestations were compared to those who did not exhibit these complications (Table 3).

### Haplotype analyses of *APRIL* and *BAFFR*

Moreover, we compared haplotype frequencies of both *APRIL* and *BAFFR* between patients with IgAV and healthy controls as well as between patients with IgAV stratified according to the specific clinical characteristics of the disease mentioned above.

The haplotype analysis of *APRIL* and *BAFFR* did not yield additional information since haplotypes frequencies of both genes were similar in patients with IgAV when compared to healthy controls (Table 4). In addition, no statistically significant differences in *APRIL* and *BAFFR* haplotype frequencies were disclosed when patients with IgAV were stratified according to the age at disease onset or to the presence/absence of GI or renal manifestations (Supplementary Table S1, Supplementary Table S2 and Supplementary Table S3 online).

**Table 4:** Haplotype analysis of *APRIL* and *BAFFR* between patients with IgAV and healthy controls.

*APRIL* haplotypes		p	OR [95% CI]
rs11552708	rs6608		
G	C	–	Ref.
A	T	0.90	0.98 [0.73–1.31]
G	T	0.20	0.77 [0.50–1.17]
A	C	0.46	0.72 [0.26–1.79]

*BAFFR* haplotypes		p	OR [95% CI]
rs7290134	rs77874543		
A	G	–	Ref.
G	G	0.57	0.93 [0.73–1.19]
G	C	0.96	1.01 [0.72–1.39]
A	C	0.16	2.08 [0.62–6.98]

IgAV: IgA vasculitis; OR: odds ratio; CI: confidence Interval.

## Discussion

Inflammatory diseases are pathologies that share common pathogenic molecular mechanisms^24,25^. With this respect, cumulative knowledge clearly suggest that BAFF, APRIL and BAFF-R are key molecules involved in the development of B-lymphocytes^1–5^, that also play a relevant role in the pathogenic processes underlying immune-mediated disorders^6–8^.

Taking into account these considerations, we aimed to determine whether *BAFF, APRIL* and *BAFFR* represent novel genetic risk factors for the pathogenesis of IgAV, a predominantly B-lymphocyte inflammatory leukocytoclastic vasculitis. For that purpose, we analysed a *BAFF* genetic variant (rs374039502), which colocalizes with the *BAFF* GCTGT>A insertion/deletion variant previously described as a common *locus* for the susceptibility to several autoimmune diseases^9,12^, in the largest series of Caucasian patients diagnosed with IgAV ever assessed for genetic studies. Interestingly, this functional *BAFF* variant is an insertion-deletion in which five nucleotides are replaced by one (GCTGT>A), being this A the risk allele that creates a shorter 3´ UTR transcript, lacking a microRNA binding site, leading to higher levels of soluble BAFF, which in turn up-regulates humoral immunity^9^. Additionally, we also assessed two tag variants within *APRIL* and *BAFFR*, which cover most of the variability of both genes, in our cohort of patients with IgAV. Our results showed no influence of *BAFF, APRIL* and *BAFFR* on the susceptibility to IgAV. Since an association between different genetic variants and an increased risk of nephritis or GI disease was disclosed in IgAV^26–29^, we also evaluated whether *BAFF, APRIL* and *BAFFR* may be related to the increased risk of nephritis or GI complications in our patients with IgAV. However, data derived from our study do not support a role of *BAFF, APRIL* and *BAFFR* polymorphisms (assessed independently or combined conforming haplotypes) in the phenotype expression of IgAV, indicating that these genes do not represent risk factors for the severity of the disease.

A previous genetic study evaluated the potential involvement of *BAFF* rs374039502 on the susceptibility to and clinical expression of giant cell arteritis (GCA), another primary systemic vasculitis that, unlike IgAV, involves large and middle-sized blood vessels^30^. Additionally, the role of this polymorphism on the pathogenesis of systemic sclerosis (SSc) was analysed in that study^30^. Nevertheless, and in keeping with our data, a lack of association of *BAFF* rs374039502 with the susceptibility and severity of GCA and SSc were reported by the authors^30^.

In summary, based on a large series of Caucasian patients, our results suggest that *BAFF, APRIL* and *BAFFR* genes do not contribute to the genetic network underlying IgAV.

## Supplementary Information

Supplementary Information.
