# Author Correction: *BAFF*, *APRIL* and *BAFFR* on the pathogenesis of Immunoglobulin-A vasculitis

**DOI:** 10.1038/s41598-021-96114-z

**Published:** 2021-08-16

**Authors:** Diana Prieto-Peña, Fernanda Genre, Sara Remuzgo-Martínez, Verónica Pulito-Cueto, Belén Atienza-Mateo, Javier Llorca, Belén Sevilla-Pérez, Norberto Ortego-Centeno, Leticia Lera-Gómez, María Teresa Leonardo, Ana Peñalba, Javier Narváez, Luis Martín-Penagos, Emilio Rodrigo, José A. Miranda-Filloy, Luis Caminal-Montero, Paz Collado, Javier Sánchez Pérez, Diego de Argila, Esteban Rubio, Manuel León Luque, Juan María Blanco-Madrigal, Eva Galíndez-Agirregoikoa, Oreste Gualillo, Javier Martín, Santos Castañeda, Ricardo Blanco, Miguel A. González-Gay, Raquel López-Mejías

**Affiliations:** 1 Research Group on Genetic Epidemiology and Atherosclerosis in Systemic Diseases and in Metabolic Bone Diseases of the Musculoskeletal System, IDIVAL, Division of Rheumatology, Hospital Universitario Marqués de Valdecilla, Avenida Cardenal Herrera Oria s/n, 39011 Santander, Spain; 2 `López Albo´ Post-Residency Programme, Hospital Universitario Marqués de Valdecilla, Santander, Spain; 3 Epidemiology and Computational Biology Department, School of Medicine, Universidad de Cantabria, and CIBER Epidemiología y Salud Pública (CIBERESP), Santander, Spain; 4 Division of Paediatrics, Hospital Universitario San Cecilio, Granada, Spain; 5 Medicine Department, Universidad de Granada, Granada, Spain; 6 Division of Paediatrics, Hospital Universitario Marqués de Valdecilla, Santander, Spain; 7 Division of Rheumatology, Hospital Universitario de Bellvitge, Barcelona, Spain; 8 Division of Nephrology, Hospital Universitario Marqués de Valdecilla, IDIVAL-REDINREN, Santander, Spain; 9 Division of Rheumatology, Hospital Universitario Lucus Augusti, Lugo, Spain; 10 Internal Medicine Dept., Hospital Universitario Central de Asturias, Instituto de Investigación Sanitaria del Principado de Asturias (ISPA), Oviedo, Spain; 11 Division of Rheumatology, Hospital Universitario Severo Ochoa, Madrid, Spain; 12 Division of Dermatology, Hospital Universitario de La Princesa, Madrid, Spain; 13 Division of Rheumatology, Hospital Universitario Virgen del Rocío, Sevilla, Spain; 14 Division of Rheumatology, Hospital Universitario de Basurto, Bilbao, Spain; 15 SERGAS (Servizo Galego de Saude) and IDIS (Instituto de Investigación Sanitaria de Santiago), NEIRID Lab (Neuroendocrine Interactions in Rheumatology and Infammatory Diseases), Research Laboratory 9, Hospital Clínico Universitario de Santiago, Santiago de Compostela, Spain; 16 Instituto de Parasitología y Biomedicina ‘López-Neyra’, CSIC, PTS Granada, Granada, Spain; 17 Rheumatology Department, Hospital Universitario de La Princesa, IIS-Princesa, Madrid, Spain; 18 School of Medicine, Universidad de Cantabria, Santander, Spain; 19 Cardiovascular Pathophysiology and Genomics Research Unit, School of Physiology, Faculty of Health Sciences, University of the Witwatersrand, Johannesburg, South Africa

Correction to: *Scientific Reports* 10.1038/s41598-021-91055-z, published online 01 June 2021

The original version of this Article contained an error in Affiliation 10, which was incorrectly given as ‘Division of Rheumatology, Hospital Universitario Central de Asturias, Instituto de Investigación Sanitaria del Principado de Asturias (ISPA), Oviedo, Spain’. The correct affiliation is listed below:

Internal Medicine Dept., Hospital Universitario Central de Asturias, Instituto de Investigación Sanitaria del Principado de Asturias (ISPA), Oviedo, Spain

The original Article has been corrected.

