# Supplementary material for: BAFF, APRIL and BAFFR on the pathogenesis of Immunoglobulin-A vasculitis
Source: Sci Rep. 2021 Jun 1;11:11510. doi: 10.1038/s41598-021-91055-z (PMC8169776; doi:10.1038/s41598-021-91055-z)
Supplement: Supplementary file 1 — Supplementary Information. [file 41598_2021_91055_MOESM1_ESM.pdf]

***BAFF*, *APRIL* and *BAFFR* on the pathogenesis of Immunoglobulin-A vasculitis**

Diana Prieto-Peña[1*], Fernanda Genre[1*], Sara Remuzgo-Martínez[1*], Verónica Pulito-Cueto[1], Belén Atienza-Mateo[1,2], Javier Llorca[3], Belén Sevilla-Pérez[4], Norberto Ortego-Centeno[5], Leticia Lera-Gómez[1], María Teresa Leonardo[6], Ana Peñalba[6], Javier Narváez[7], Luis Martín-Penagos[8], Emilio Rodrigo[8], José A. Miranda-Filloy[9], Luis Caminal-Montero[10], Paz Collado[11], Javier Sánchez Pérez[12], Diego de Argila[12], Esteban Rubio[13], Manuel León Luque[13], Juan María Blanco-Madrigal[14], Eva Galíndez-Agirregoikoa[14], Oreste Gualillo[15], Javier Martín[16], Santos Castañeda[17], Ricardo Blanco[1], Miguel A. González-Gay[1, 18, 19 §], Raquel López-Mejías[1 §*]

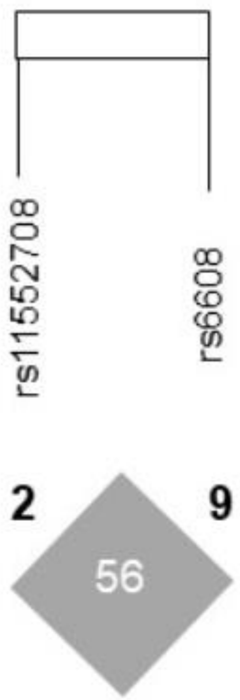

Supplementary Fig. 1. Linkage disequilibrium of the *APRIL* polymorphisms (in European population) measured by r^2 coefficient. Data obtained by 1000 Genomes Project and Haploview v.4.2 software, considering the r^2 threshold set at 0.8 and minimum minor allele frequency at 0.05.

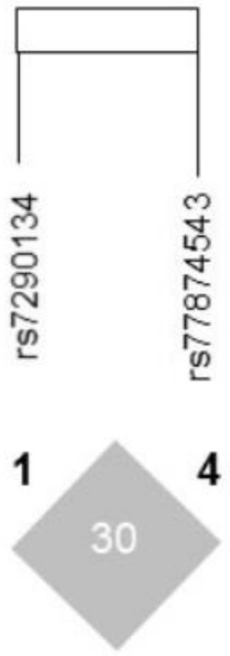

Supplementary Fig. 2. Linkage disequilibrium of *BAFFR* polymorphisms (in European population) measured by r^2 coefficient. Data obtained by 1000 Genomes Project and Haploview v.4.2 software, considering the r^2 threshold set at 0.8 and minimum minor allele frequency at 0.05.

Supplementary Table S1. Haplotype analysis of *APRIL* and *BAFFR* between patients with IgAV stratified according to the age at disease onset.

APRIL haplotypes		p	OR [95% CI]
rs11552708	rs6608		
G	C	-	Ref.
A	T	0.64	1.15 [0.63-2.25]
G	T	0.09	2.71 [0.82-14.03]
A	C	0.61	0.65 [0.11-6.95]

BAFFR haplotypes		p	OR [95% CI]
rs7290134	rs77874543		
A	G	-	Ref.
G	G	0.33	0.79 [0.48-1.32]
G	C	0.79	0.92 [0.47-1.91]
A	C	0.11	0.31 [0.05-2.17]

IgAV: IgA vasculitis; OR: Odds Ratio; CI: confidence Interval.

Supplementary Table S2. Haplotype analysis of *APRIL* and *BAFFR* between patients with IgAV stratified according to the presence/absence of GI manifestations.

APRIL haplotypes		p	OR [95% CI]
rs11552708	rs6608		
G	C	-	Ref.
A	T	0.64	1.12 [0.68-1.83]
G	T	0.18	1.64 [0.76-3.69]
A	C	0.33	2.23 [0.36-23.59]

BAFFR haplotypes		p	OR [95% CI]
rs7290134	rs77874543		
A	G	-	Ref.
G	G	0.46	1.16 [0.76-1.77]
G	C	0.90	1.03 [0.60-1.79]
A	C	0.33	2.21 [0.36-23.39]

IgAV: IgA vasculitis; GI: gastrointestinal; OR: Odds Ratio; CI: confidence Interval.

Supplementary Table S3. Haplotype analysis of *APRIL* and *BAFFR* between patients with IgAV stratified according to the presence/absence of renal manifestations.

APRIL haplotypes		p	OR [95% CI]
rs11552708	rs6608		
G	C	-	Ref.
A	T	0.12	0.67 [0.39-1.13]
G	T	0.97	0.99 [0.44-2.11]
A	C	0.82	1.20 [0.17-7.13]
BAFFR haplotypes		p	OR [95% CI]
rs7290134	rs77874543		
A	G	-	Ref.
G	G	0.61	0.90 [0.58-1.37]
G	C	0.56	0.85 [0.48-1.49]
A	C	0.59	0.54 [0.01-6.84]

IgAV: IgA vasculitis; OR: Odds Ratio; CI: confidence Interval.
